# Amino acid changes within the Bunyamwera virus nucleocapsid protein differentially affect the mRNA transcription and RNA replication activities of assembled ribonucleoprotein templates

**DOI:** 10.1099/vir.0.024240-0

**Published:** 2011-01

**Authors:** Cheryl T. Walter, Diana F. Costa Bento, Ana Guerrero Alonso, John N. Barr

**Affiliations:** Institute for Molecular and Cellular Biology, Faculty of Biological Sciences, University of Leeds, Leeds LS2 9JT, UK

## Abstract

The genome of Bunyamwera virus (BUNV) comprises three RNA segments that are encapsidated by the virus-encoded nucleocapsid (N) protein to form ribonucleoprotein (RNP) complexes. These RNPs are the functional templates for RNA synthesis by the virus-encoded RNA-dependent RNA polymerase (RdRp). We investigated the roles of conserved positively charged N-protein amino acids in RNA binding, in oligomerization to form model RNPs and in generating RNP templates active for both RNA replication and mRNA transcription. We identified several residues that performed important roles in RNA binding, and furthermore showed that a single amino acid change can differentially affect the ability of the resulting RNP templates to regulate the transcription and replication activities of the RdRp. These results indicate that the BUNV N protein possesses functions outside of its primary role of RNA encapsidation.

The family *Bunyaviridae* of segmented negative-strand (SNS) RNA viruses contains more than 350 members categorized into five genera: *Orthobunyavirus, Hantavirus, Nairovirus, Phlebovirus* and *Tospovirus*. The prototype of the genus *Orthobunyavirus* is Bunyamwera virus (BUNV), which serves as a model for studying important pathogens within this family. As with all bunyaviruses, the BUNV genome comprises three segments, named small (S), medium (M) and large (L) (Elliott, 1990). These strands are the templates for two fundamentally distinct processes, namely RNA replication and mRNA transcription. In common with other negative-stranded RNA viruses, the active template for these processes is not naked RNA, but is instead the RNA genome bound with virally encoded nucleocapsid (N) protein to form a ribonucleoprotein (RNP) complex. N protein remains associated with replication products throughout the infectious cycle, and thus the N–RNA interaction is critical for virus viability.

Several studies have investigated the specificity of this interaction for bunyaviruses. They indicate that, while there is no obligatory encapsidation sequence, there is evidence of preferred binding for viral sequences (Mir & Panganiban, 2005; Mohl & Barr, 2009; Ogg & Patterson, 2007; Osborne & Elliott, 2000). It has been proposed that this property may allow selective encapsidation of viral rather than host cell RNAs, and thus selective packaging of only viral RNPs into progeny virions. In contrast, molecular details of the role of the N protein in N–RNA interactions are lacking currently. Here, we have analysed for the first time the roles of individual BUNV N-protein amino acids in binding the RNA template. In addition, we have examined how these roles relate to both RNP assembly and to formation of the active template for both mRNA transcription and RNA replication.

Previous structural studies with human respiratory syncytial virus, rabies virus and vesicular stomatitis virus (VSV) show that the mechanism of RNA binding within the non-segmented negative-strand (NNS) RNA-virus RNP relies on multiple interactions, mediated by a variety of residues within specialized RNA-binding grooves (Albertini *et al.*, 2006; Green *et al.*, 2006; Tawar *et al.*, 2009). In all three RNPs, positively charged amino acid side-chains interact with the RNA–phosphate backbone. Similar involvement of charged residues in RNA binding has also been described for the influenza virus nucleoprotein, and the crystal structure reveals a potential RNA-binding groove lined with conserved positively charged residues (Elton *et al.*, 1999; Ye *et al.*, 2006). Because of the important role of positively charged amino acids in RNA binding in the context of both viral RNPs and also in general (Terribilini *et al.*, 2006), in this current study we focused exclusively on these residues.

The BUNV N protein contains 31 positively charged amino acids. To identify those likely to possess critical functions, we aligned N proteins from eight orthobunyaviruses (Supplementary Fig. S1, available in JGV Online). We selected 15 of these that were best conserved in terms of identity or positive charge for further analysis. We analysed the role of these conserved residues in RNA binding by exploiting our previously described ability to express and purify soluble N protein from bacterial cells as an N–RNA complex (Rodgers *et al.*, 2006). When purified, the BUNV N protein formed homogeneous and stable soluble assemblies in which N-protein tetramers bound 48 nt sections of bacterial-cell RNA, forming model RNPs (Mohl & Barr, 2009). The establishment of this N–RNA assembly system allowed us to introduce mutations into model RNPs and assess how these changes affected RNA-binding ability.

Previously described plasmid pET-BUN-N (Rodgers *et al.*, 2006) was modified by using PCR-mediated site-directed mutagenesis to encode 15 N-protein variants incorporating single alanine substitutions at each of the 15 conserved positively charged residues (Supplementary Fig. S1). Briefly, these altered N proteins were expressed in bacteria as N-terminally histidine-tagged fusion proteins and then purified by using affinity chromatography. To test whether the N-protein alterations affected expression, stability and folding, purified N proteins were analysed by using circular dichroism (CD) and SDS-PAGE at various time points post-expression (data not shown). This analysis showed that all altered proteins were expressed at an abundance indistinguishable from wild type. In addition, with the exception of K166A, all proteins were correctly folded and highly stable. In contrast, K166A displayed a somewhat-disordered CD spectrum, and, following several days of incubation at 4 °C, was found to dissociate into two polypeptide fragments at the approximate point of alteration. To allow the functional comparison of all altered proteins, analysis of RNA binding and oligomerization was performed immediately following purification.

RNA was harvested from RNPs by phenol–chloroform and chloroform extractions from an equal mass of protein (Fig. 1a), which was then visualized by native agarose-gel electrophoresis (Fig. 1b) and ethidium-bromide staining, as previously described (Mohl & Barr, 2009). We identified three N-protein mutants with RNA-binding abilities significantly different from wild type. The most striking was R94A, for which no RNA binding was detected. In addition, RNA binding of mutants R40A and K50A was reduced significantly. All other mutants displayed RNA binding that was comparable to wild type.

As RNA binding of the 48-mer RNA species relies on N-protein tetramer formation, it was also possible that changes in RNA binding reflected changes in multimerization. Therefore, we used size-exclusion chromatography to assess the oligomeric state of wild-type and mutant N proteins, as previously described (Mohl & Barr, 2009). Purified N proteins were analysed by using size-exclusion chromatography with a Superdex S200 column (GE Healthcare Life Sciences) immediately following purification and their elution profile determined by spectroscopy and compared with molecular mass standards (Fig. 2).

As expected from previous results (Mohl & Barr, 2009), wild-type N protein was eluted predominantly as a tetramer with an observed molecular mass of 113.7 kDa (115.1 kDa predicted) with a minor peak at 27.2 kDa corresponding to monomer (28.8 kDa predicted) (Fig. 2a). Analysis of R94A (Fig. 2a) indicated the quantity of monomer was increased, such that monomers and tetramers were equally abundant. However, a major fraction of the expressed material was in the tetramer form, an observation supported by native PAGE analysis of purified R94A protein (not shown). Therefore, while the presence of monomers may contribute to the loss of R94A RNA-binding ability, the lack of detectable bound RNA in R94A tetramers suggested that this residue played an important role in RNA binding. Consistent with this functional assignment, recent experiments performed in the Elliott laboratory show that infectious viruses bearing the R94A mutation could not be rescued (Eifan & Elliott, 2009), revealing that R94 plays a critical role in the BUNV life cycle. The results of our analysis indicate that this critical role may be RNA binding.

The elution profiles of R40A and K50A revealed no significant change in monomer/tetramer ratios, indicating that their reduced RNA binding was not because of reduced multimerization (Fig. 2b). Interestingly, both mutants showed an increased proportion of faster-eluting species compared with wild type, with masses >1000 kDa. Similar large complexes were also detected for K55A, R101A and K179A, all of which displayed wild-type levels of RNA binding (Fig. 2c), indicating that formation of such higher-order structures did not correlate with reduced RNA binding. Therefore, these data suggested that R40 and K50 contribute to RNA binding, although they are not essential for it. Interestingly, both these mutants have previously been rescued into infectious viruses (Eifan & Elliott, 2009), although the resulting viruses exhibited considerably attenuated growth characteristics. As described above for residue R94, our findings, reported here, that R40A and K50A are deficient in RNA binding offer a plausible explanation for why the resulting rescued viruses are either non-viable or growth deficient, consistent with the important role of RNA binding in the virus life cycle.

All of the other N-protein mutants exhibited monomer/tetramer ratios that were indistinguishable from wild-type N protein (data not shown), and so we concluded that none of these residues play a critical role in N protein oligomerization.

We analysed the ability of all 15 N mutants to support the assembly of BUNV RNP templates, which were active for both transcription and replication. This was achieved by using a previously described assay (Barr *et al.*, 2003; Dunn *et al.*, 1995) that directly detects RNAs generated by the BUNV RNA-dependent RNA polymerase (RdRp) from assembled RNP templates. Briefly, plasmids expressing model segment BUN-M(ren) and the BUNV-S and -L ORFs were transfected into BHK-21 cells that had previously been infected with vaccinia recombinant vTF7-3, which expresses T7 RNA polymerase. BUNV-specific RNAs generated from the resulting RNPs were harvested and detected by primer extension analysis by using ^33^P-end-labelled oligonucleotide REN-PAGE, which was designed to anneal to positive-sense products of both transcription and replication. To assess the role of the 15 charged residues in the formation and activity of RNPs, the plasmid expressing the BUNV N-protein ORF was modified to incorporate each amino acid change individually.

Primer-extension analysis detected both mRNAs and anti-genomic RNAs generated from assembled RNPs. By using densitometry to measure the abundance of their corresponding products, we quantitatively assessed the role of each altered residue on transcription and replication activities (Fig. 3). Most mutations had a minimal effect on RNP activity, such that levels of replication and transcription were each comparable to those of the wild type. However, mutation R94A exhibited a striking phenotype, which was that it showed dramatically reduced transcriptional activity despite exhibiting robust replication. As described above, the R94A mutation cannot be rescued into infectious virus (Eifan & Elliott, 2009), and our results presented here suggest that the dramatically reduced transcriptional ability may be responsible.

We are curious as to how the N protein is able to impart differential effects to the RdRp functions of transcription and replication. Presumably, any mechanism responsible is unlikely to involve activities that are common to both these RNA synthesis modes. One possibility is that the R94A mutation somehow affects access of the RdRp to *cis*-acting signals for replication versus transcription. BUNV-replication and -transcription signals comprise interacting nucleotides from both ends of each active RNP template, although the critical nucleotides that comprise each signal are distinct (Barr *et al.*, 2003, 2005; Barr & Wertz, 2004, 2005). Perhaps the N protein differentially affects the accessibility of these nucleotides to the BUNV RdRp by altering the exposure of the RNA bases to the polymerase. It is also possible that alterations in base exposure may affect how the ends of the template interact, which also affects RdRp function. Another interesting possibility is that BUNV-replication and -transcription activities may be performed by distinct complexes, as has been proposed for VSV (Gupta *et al.*, 2003; Qanungo *et al.*, 2004), and differences in RNP composition may differentially affect recruitment of these complexes to the template.

The concept that the N protein of a negative-stranded RNA virus can differentially modulate the template activity of the RNP is not new, having been established with the polR1 mutant of the NNS RNA virus VSV (Chuang & Perrault, 1997). More recently, this concept was reinforced by the identification of additional N-protein residues that differentially affected VSV RNP activity, which was guided by the high-resolution N–RNA structure (Harouaka & Wertz, 2009; Nayak *et al.*, 2009; Rainsford *et al.*, 2010). However, the RNPs of SNS RNA viruses such as BUNV exhibit important differences from those of the NNS RNPs, including increased sensitivity to RNase digestion, which implies increased exposure of the RNA backbone on the external surface of the RNP. Our results indicate that despite this less intimate N–RNA association, BUNV N-protein residues are likely to exert a major influence on how the RdRp recognizes the RNA template. Taken together, these results show that N proteins of both NNS and SNS RNA viruses possess critical functions outside their roles of RNA encapsidation.

## Supplementary Material

[Supplementary Figure]

## Figures and Tables

**Fig. 1.**
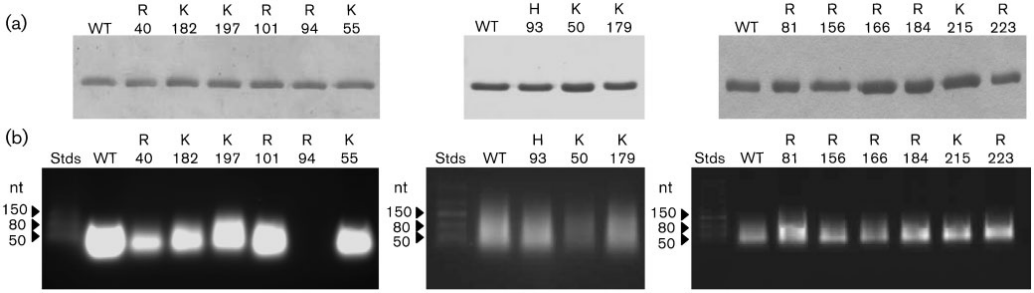
Analysis of the RNA-binding ability of BUNV N-protein mutants. (a) The high purity of BUNV N-protein samples used to extract RNA was confirmed by subjecting purified proteins to SDS-PAGE analysis and visualization by using Coomassie staining immediately prior to RNA extraction. (b) RNA was extracted from an equal mass of mutant N protein expressed in bacterial cells, electrophoresed on a non-denaturing agarose gel and visualized by ethidium-bromide staining. Each N protein mutant is identified with the number of the corresponding altered residue. RNA size markers are shown by arrowheads. Stds, Standards; WT, wild type.

**Fig. 2.**
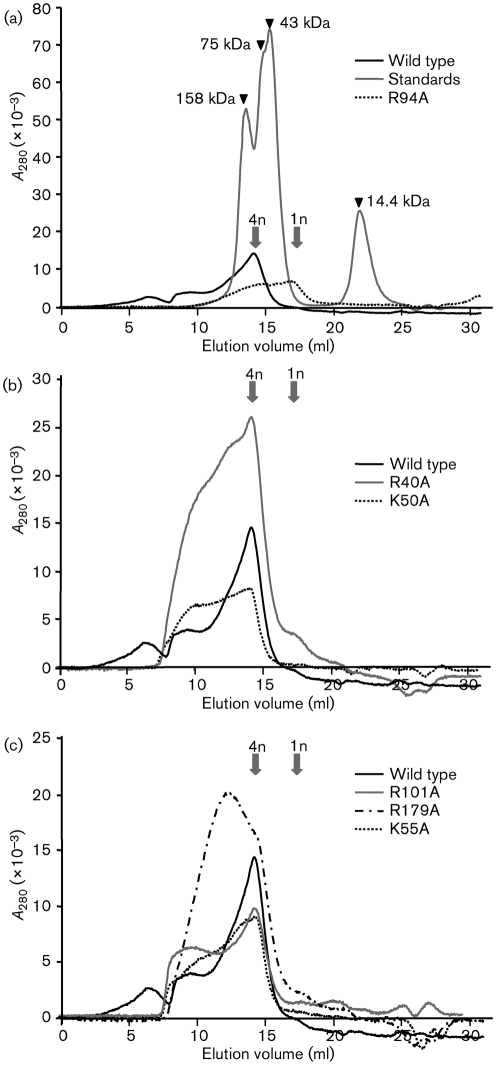
Analysis of the ability of N-protein mutants to multimerize. Purified BUNV N-protein mutants were analysed by size-exclusion chromatography and elution profiles compared with both wild-type N protein and molecular mass standards. Elution positions of N-protein monomers (1n) and tetramers (4n) are marked with arrows.

**Fig. 3.**
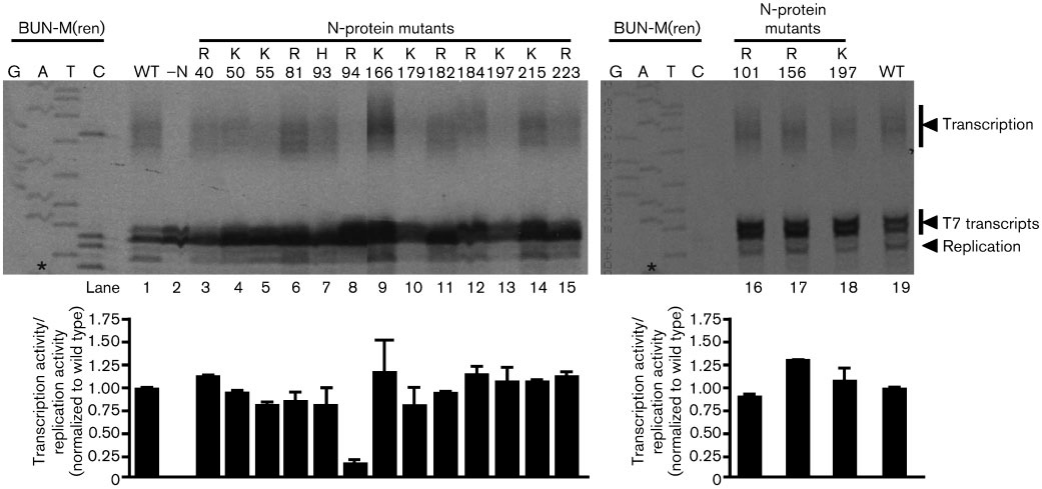
RNA replication and mRNA transcriptional abilities of N-protein mutants were examined by reconstituting BUNV RNPs within BHK-21 cells. Lanes are marked with the number of the altered residue of the mutant used to assemble the corresponding RNPs. RNAs were harvested and analysed by primer extension, which detected T7 primary transcripts, positive-sense replication products and mRNAs. The cDNA encoding RNA template BUN-M(ren) was sequenced with the same primer to act as a size marker. *, Nucleotide corresponding to the anti-genomic 5′ end. Autoradiographs from two experiments were scanned and analysed by densitometry, and the RNA synthesis characteristics of each RNP were expressed as a ratio of transcriptional activity/replication activity, with each ratio normalized to wild type and which is shown below each corresponding lane. Error bars (sem) are shown.
