# Supplementary material for: Amino acid changes within the Bunyamwera virus nucleocapsid protein differentially affect the mRNA transcription and RNA replication activities of assembled ribonucleoprotein templates
Source: J Gen Virol. 2011 Jan;92(Pt 1):80–4. doi: 10.1099/vir.0.024240-0 (PMC3052533; doi:10.1099/vir.0.024240-0)
Supplement: [Supplementary Figure] [file supp_92_1_80__index.html]

 Amino acid changes within the Bunyamwera virus nucleocapsid protein differentially affect the mRNA transcription and RNA replication activities of assembled ribonucleoprotein templates -- Walter et al. 92 (1): 80 Data Supplement - Supplementary Figure -- Journal of General Virology 
